# Blue dot cataract with Sutural cataract: A rare association and its management

**DOI:** 10.22336/rjo.2025.19

**Published:** 2025

**Authors:** Nirupama Kasturi, Mary Stephen, Pacha Pavani, Priyanka Mohan

**Affiliations:** Department of Ophthalmology, JIPMER, Puducherry, India

**Keywords:** blue dot cataract, sutural cataract, astigmatism, phacoemulsification, developmental cataract, toric lens

## Abstract

Cataracts are one of the leading causes of reversible blindness worldwide in the paediatric population. In general, paediatric cataracts significantly impact the child’s visual development and their family, both emotionally and financially. However, certain developmental cataracts, like blue dot cataracts, are innocuous and will not cause visual morbidity. A sutural cataract involves the opacification of the anterior and posterior Y sutures, which can rarely affect vision. This is a case report of a 20-year-old male with vision loss, a rare association of blue dot cataract, sutural cataract, and significant astigmatism managed with uncomplicated phacoemulsification and Toric intra-ocular lens. Coexistence of blue dot cataracts with sutural cataracts is often rare, as is the presentation in the third decade of life with vision loss.

## Introduction

Blue dot cataract is the most common form of paediatric cataract; in most cases, it does not cause any visual impact on the patient. The overall incidence of developmental cataracts is 3 in 10,000 live births [1]. Most cases of developmental cataracts are autosomal dominant inheritance with incomplete penetrance. Characteristically, blue dot cataracts have multiple diffusely distributed blue and white dot-like opacities in the cortex, which involve the fetal nucleus. The opacities usually will not cause vision disturbance. Sutural cataract typically causes the opacification of Y sutures. Blue dot and sutural cataracts often progress through childhood and early adulthood [2]. This is a case report of a young male with significant visual disability and the presence of both blue dot and sutural cataract with significant astigmatism. The patient was managed with uncomplicated phacoemulsification and toric intra-ocular lens implantation. Blue dot cataracts with sutural cataracts are rare and present with vision loss and associated significant astigmatism is uncommon.

## Case history

**Fig. 1:**
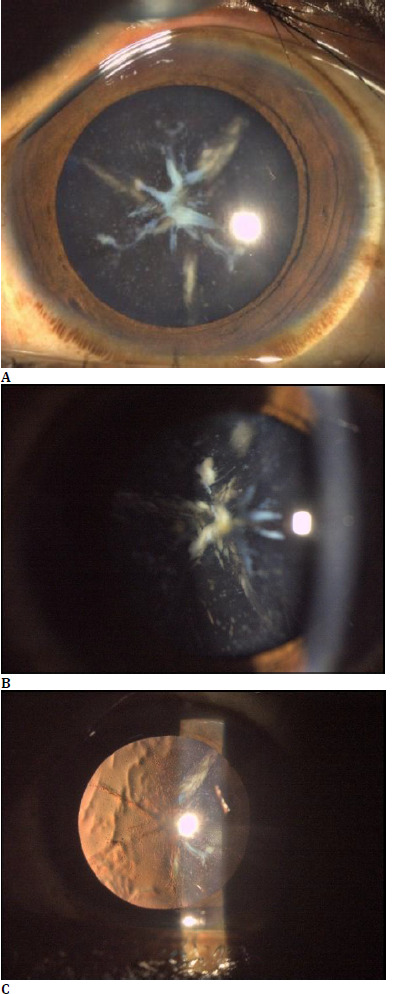
Slit lamp photo of the patient presenting classical blue and white opacities with a dense sutural cataract of the right eye (**A**. Diffuse illumination; **B**. Slit beam; **C**. Retro-illumination)

We report the case of a 20-year-old male who presented to the outpatient department with complaints of gradual onset painless diminution of vision in both eyes. The best corrected Snellen’s visual acuity of both eyes was 6/24, and refraction showed four diopters with simple myopic astigmatism in both eyes. A slit lamp examination showed a clear cornea with a normal anterior chamber. Lens examination in an undilated state revealed central sutural opacities and, on pupillary dilation, multiple breadcrumb-like blue and white dot opacities distributed in the cortex all around with dense opacification of the anterior and posterior Y sutures in both eyes (**Fig. 1**).

Areas of clear lens were noted between the opacities. Fundus examination was normal. A routine cataract pre-operative workup with optical biometry was performed. On alternate days, uneventful phacoemulsification with toric intraocular lens placement was performed on both eyes, with good post-operative recovery.

## Discussion

Developmental cataracts are one of the leading causes of blindness in both developed and developing countries [3]. The exact etiology of developmental cataracts is not known. However, genetics plays a crucial role in the causes. Multiple mutations have been studied to be causative for blue dot and sutural cataracts, which predominantly involve beta crystallins, gamma crystallins, and significant intrinsic lens fibre genes - most cases of developmental cataract present with nystagmus, strabismus, or leukocoria [4]. Early identification and prompt intervention can significantly impact the child’s mental and economic growth [4]. A few visually innocuous developmental cataracts, like blue dot cataracts, often will not cause significant visual morbidity. Blue dot cataract, also known as cerulean cataract, was first described by Vogt in 1922. The opacities may progress through adulthood and have a concentric radial appearance. Studies have presented the innocuous nature of blue dot cataracts and the rare indication for surgical intervention before adulthood. Early intervention is warranted when associated with nystagmus and amblyopia [5-7]. The association of classical blue dot cataracts with sutural cataracts that lead to visual disability is rare [2]. This was a case report of a young student with significant vision loss in both eyes, and examination revealed the presence of a classical blue dot cataract with dense sutural cataract and high with the rule of astigmatism. With uncomplicated phacoemulsification and toric intraocular lens, the patient improved very well. To our knowledge, reports on the association of blue dot cataracts with sutural cataracts are rare, and the need for their intervention has not been described.

## Conclusion

Even though developmental cataracts cause significant visual morbidity in the paediatric population, blue dot cataract is a variant with no vision loss. The authors reported a rare case of classical blue dot cataract with dense sutural cataract in a young male who was managed with uneventful cataract surgery.
